# Early Detection of Vasa Previa in a Low-Risk Pregnancy Leading to Favourable Perinatal Outcome: A Case Report

**DOI:** 10.7759/cureus.91403

**Published:** 2025-09-01

**Authors:** Kimberly Lim Xinyi, Devina Nagraj, Amit Verma

**Affiliations:** 1 Obstetrics and Gynaecology, Manchester University National Health Service (NHS) Foundation Trust, Manchester, GBR; 2 Obstetrics and Gynaecology, Topiwala National Medical College and Bai Yamunabai Laxman Nair Charitable Hospital, Mumbai, IND; 3 Obstetrics and Gynaecology, Wrightington, Wigan and Leigh National Health Service (NHS) Foundation Trust, Wigan, GBR

**Keywords:** cervical length measurement, low lying placenta, placenta previa, transvaginal ultrasound scan, vasa previa

## Abstract

Vasa previa is a condition with a high mortality rate where the foetal blood vessels traverse, or lie in proximity to, the internal cervical os and are typically not supported by the umbilical cord or placenta. Recognised risk factors of vasa previa include velamentous umbilical cord insertion, assisted reproductive technologies, multiple pregnancies, bilobed or succenturiate placentas, and low-lying placental insertion. Perinatal mortality rate remains high when vasa previa is not detected antenatally, although current evidence does not support universal screening for vasa previa in the general obstetric population. This report highlights the case of a primigravida lady in whom a succenturiate placenta and low-lying vessels were identified on ultrasound at 22 weeks' gestation. She underwent a planned caesarean section and achieved a favourable perinatal outcome. Although transvaginal ultrasound can detect vasa previa antenatally, the condition may be missed even under optimal imaging conditions; this case highlights the utility of transvaginal ultrasound in the perinatal diagnosis of vasa previa, which can significantly improve perinatal outcomes. Further research into universal vasa previa screening should be considered.

## Introduction

Vasa previa (VP) is a rare condition linked to a high foetal mortality rate when not detected antenatally [1]. This happens when the unprotected foetal blood vessels pass through the amniotic membranes and course over the cervix [2]. Vasa previa could be categorised into two types [2,3]. Type I occurs when the velamentous cord is connected to a single-lobed placenta, and the foetal vessels pass freely within the amniotic membranes and extend along the cervix, and Type II occurs when the foetal vessels connect the placenta with a succenturiate or accessory lobe [2,3]. Recently, Type III has been described as a rare form of vasa previa that occurs when aberrant foetal vessels extend from the placental surface and loop back to the placenta in a 'boomerang' pattern, without velamentous cord insertion or multilobed placenta [4,5].

Vasa previa has been estimated to occur in approximately 1 in 1200 to 1 in 5000 pregnancies, although recent studies suggest it may occur more frequently than previously considered [3]. The main risk factors of vasa previa are velamentous cord insertion and the presence of a bilobed or succenturiate placenta, with the former accounting for most cases [2]. Other risk factors include placenta previa or low-lying placenta, assisted reproductive technologies, and multiple pregnancies [3]. Around 60% of women with vasa previa at delivery had placenta previa or low-lying placenta at the second-trimester ultrasound, and approximately 20% continue to exhibit a low-lying placenta at delivery [2]. Both transabdominal and transvaginal colour Doppler imaging offer the best diagnostic reliability for vasa previa [6]. The visualisation of umbilical cord insertion during the second trimester through screening amongst women with high risk factors has improved diagnosis and management, thus decreasing the foetal mortality rate [7]. Targeted screening for women with risk factors for vasa previa is preferred, and the timing of delivery and management should be tailored according to individual risk factors [8].

A good perinatal outcome is largely dependent on prenatal diagnosis and timely Caesarean section [9,10]. A pooled analysis of seven studies including cases of vasa previa with and without prenatal diagnosis showed a perinatal survival rate of 72.1% in undiagnosed cases of vasa previa compared with 98.6% in those diagnosed antenatally, with a 25-fold higher risk of perinatal death in cases without an antenatal diagnosis of vasa previa (odds ratio [OR], 25.39; 95% CI, 7.93-81.31; p < 0.0001) [10]. Similarly, the risk of hypoxic morbidity was markedly elevated up to 50-fold in cases with undiagnosed vasa previa compared with those diagnosed prenatally (36/61 vs 5/224; OR, 50.09; 95% CI, 17.33-144.79) [10]. Elective caesarean section should be performed for such women before the onset of labour in the third trimester [6]. The Royal College of Obstetricians and Gynaecologists (RCOG) has suggested that elective caesarean delivery at a gestation of 34 to 36 weeks is reasonable for asymptomatic women with a prenatal diagnosis of vasa previa [6].

This case report presents a lady in her late 20s (G1, P0) who was diagnosed with vasa previa at 23 weeks, followed by an elective caesarean section at 35+5 weeks with a good perinatal outcome.

## Case presentation

**Figure 1:**
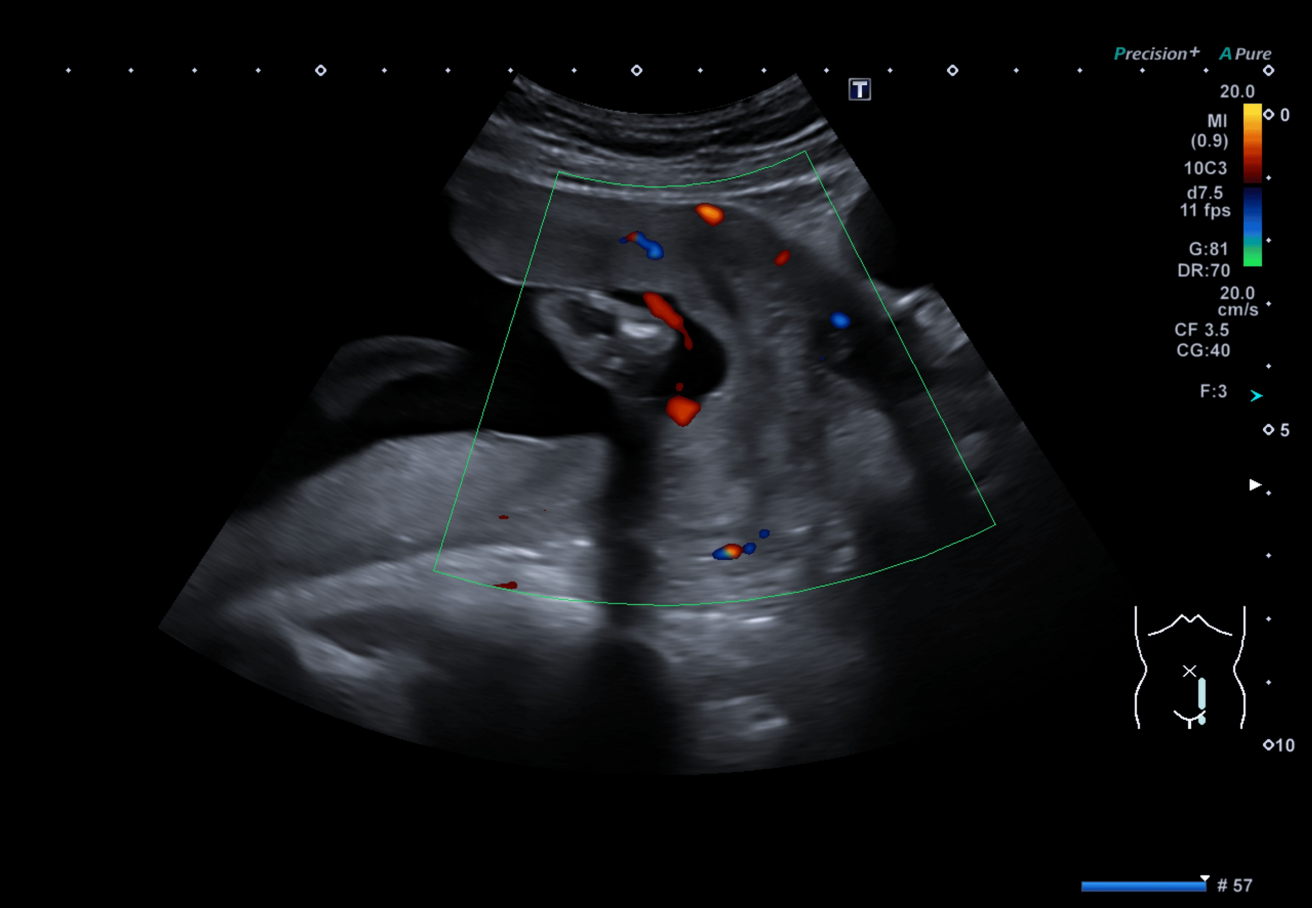
Routine anomaly scan (FAS) at 22+1 weeks showing low placenta with vasa previa

The patient, a primigravida lady in her late 20s, was booked by midwives around 10 weeks of gestation. She has a past medical history of generalised anxiety disorder and longstanding palpitations, leading to a diagnosis of sinus tachycardia after thorough investigations. She was followed up in the joint obstetric cardiac clinic regarding this and managed with low-dose bisoprolol from 21 weeks of gestation due to worsening symptoms. The dating scan and all the booked blood tests were normal. She is Rhesus O negative. She had a routine foetal anomaly scan (FAS) at 20+1 weeks. As the initial FAS was incomplete due to foetal position, this was followed by a repeat FAS at 22+1 weeks of gestation for completion (Figure 1). This showed a low-lying right lateral placenta with an anterior or left succenturiate lobe and evidence of bridging vessels in some sections on the transvaginal colour Doppler ultrasound, raising the suspicion of vasa previa. She was referred for a foetal medicine scan at 23+1 weeks. The transvaginal scan showed a posterior low-lying placenta reaching the internal cervical os with a succenturiate lobe anteriorly and slightly towards the left side. Umbilical vessels, unsupported by either the umbilical cord or the placental tissue, traversed the foetal membranes of the lower segment above the cervix over the internal os, thus confirming a diagnosis of vasa previa. A paediatric referral was sent accordingly. At 24 weeks of gestation, she experienced mild per vaginum (PV) bleeding, which was managed appropriately with an in-utero transfer to a tertiary neonatal unit after optimisation with steroids and magnesium sulphate for neuroprotection. After two days in inpatient stay, the bleeding settled, and she was followed up as an outpatient in the local foetal medicine unit with fortnightly cervical length scans.

**Figure 2:**
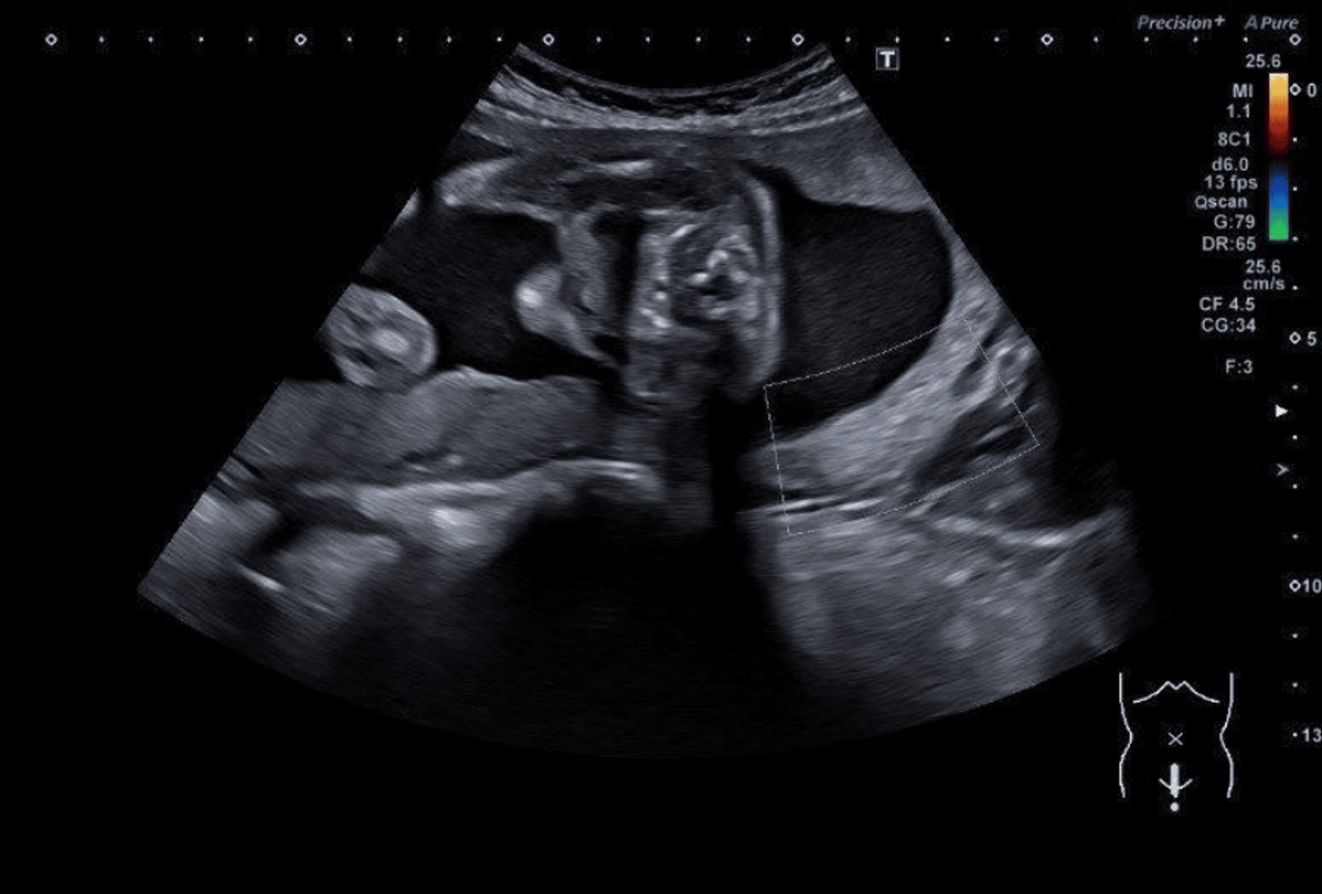
A scan at 23+1 weeks showed a posterior low-lying placenta reaching the internal cervical os with a succenturiate lobe anteriorly and slightly towards the left side

**Figure 3:**
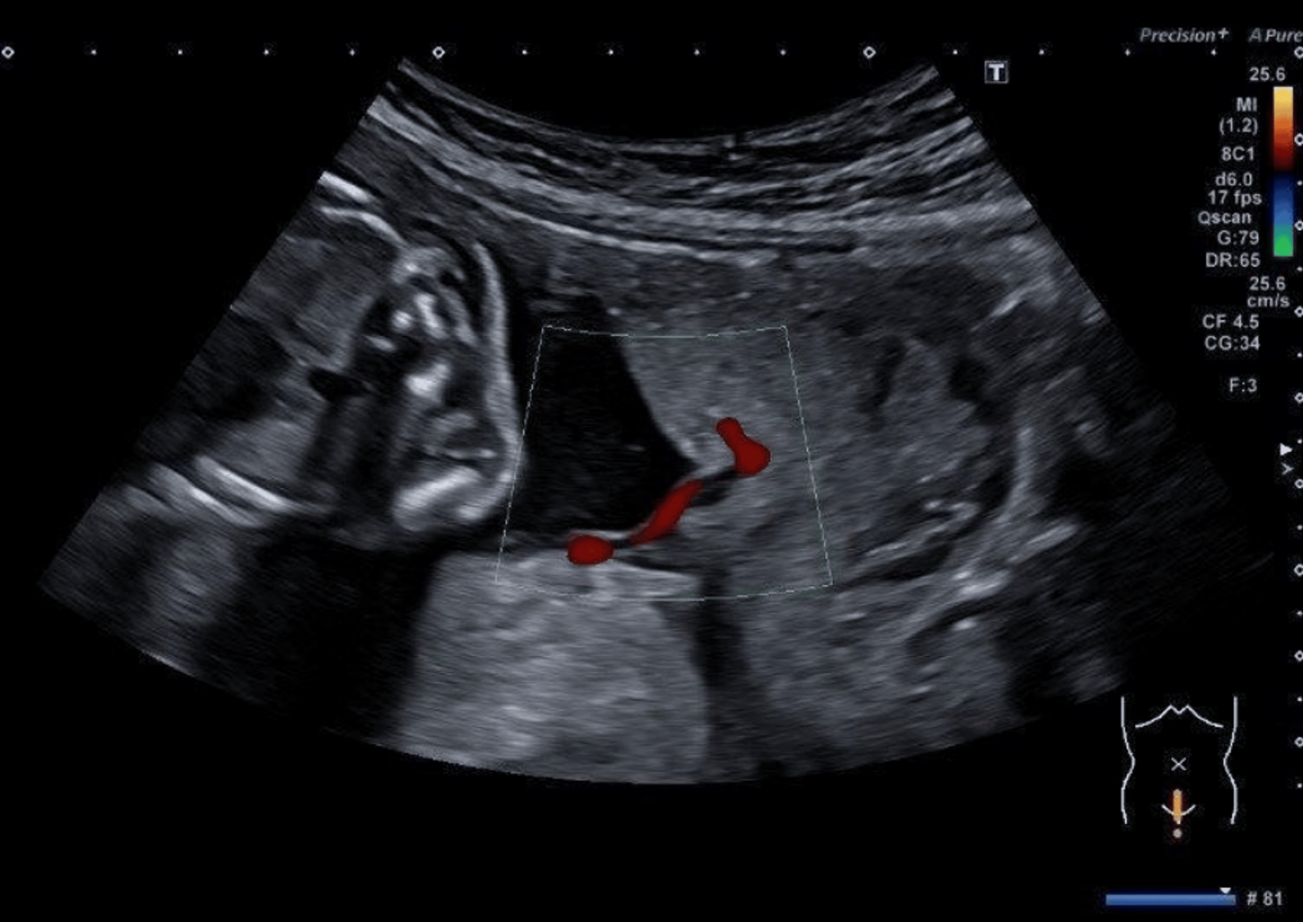
Vasa previa at 23+1 weeks: the foetal vessels run through the free placental membranes

The scan at 23+1 weeks reported the placenta was lying low, reaching the internal cervical os with a succenturiate lobe anteriorly and slightly towards the left side (Figure 2). Umbilical vessels, unsupported by either the umbilical cord or placental tissues, were seen traversing the foetal membranes in the lower segment above the cervix, indicating vasa previa (Figure 3). Cervical length was 4 cm with no evidence of funnelling. As per the foetal medicine advice, she had serial scans fortnightly after 26 weeks to monitor the cervical length. She was also advised for hospitalisation in case of any evidence of cervical shortening less than 2.5 cm due to a higher risk of preterm labour [11]. An ultrasound scan at 26+1 weeks showed a low-lying placenta reaching the internal cervical os with an anterior succenturiate lobe and evidence of vasa praevia, with an estimated foetal weight (EFW) of 872 g (50th centile), normal liquor volume, and Umbilical artery (UA) Doppler Pulsatility Index [11]. The cervical length was 3.1cm with no funneling. This was followed by a growth scan at 28+1 weeks of gestation, which showed an EFW of 1029 g and normal liquor volume and UA Doppler, decreased growth velocity at the 30th centile, and a daily weight gain of 11.2 g. Evidence of vasa previa was seen as before. Cervical length was 3.8 cm with no funnelling. The scan at 31 weeks showed normal growth velocity with an EFW of 1584 g (40th centile) with a daily weight gain of 26.4 g. The liquor and UA Doppler were normal, and the cervical length measured 3.2 cm [11]. At 32 weeks, the scan showed normal liquor and Doppler with a low-lying placenta and an anterior succenturiate lobe lying 17 mm away from the internal cervical os. It was a technically difficult scan due to the foetal head being too close to the cervix. No obvious vessels were noted over the cervix. The cervix was 3.3cm and closed. At 34 weeks, the growth scan showed an EFW of 2142 g (45th centile), with normal liquor and UA Doppler, and a normal cervical length of 3.6 cm [11]. The placenta appeared right lateral with an anterior succenturiate lobe, as no vessels were identified in the region of the cervix on this scan. Table 1 shows the ultrasound assessments.

**Table 1:** Serial ultrasound assessments of foetal growth and cervical length

Scan at gestation (weeks)	Estimated fetal weight (g) and centile	Cervical length (cm)
26	872g (50th centile)	3.1
28+1	1029g (30th centile)	3.8
31	1584g (40th centile)	3.2
34	2142g (45th centile)	3.6

She was admitted electively at approximately 33 weeks for reassurance as per maternal request, followed by a successful elective C-section at 35+5 weeks of gestation [6]. She was given a course of steroids prior to her caesarean section to reduce the risk of neonatal respiratory distress and the need for neonatal unit admission. The postoperative course for the mother was uneventful. The baby was born at the 21.8th centile with a birthweight of 2200 g. The Apgar scores were 9, 10, and 10 at 1, 5, and 10 minutes, respectively. The baby developed hypoglycaemia, likely related to antenatal steroid use, which was managed conservatively with feeds, and both mother and baby were discharged safely after seven days.

## Discussion

Vasa previa is a rare but life-threatening condition if not diagnosed and managed in a timely manner. It can lead to complications including haemorrhage, preterm delivery, the need for emergency caesarean section, foetal growth restriction, foetal distress, admission to neonatal intensive care units, and increased perinatal morbidity and mortality rates [12]. One study reports an overall perinatal mortality rate of 35.5% (55/155), with outcomes markedly improved when vasa previa was diagnosed antenatally (3.3% vs 56.4%, p<0.001) [12]. Multiple studies have demonstrated that prenatal detection of vasa previa is critical to reducing perinatal morbidity and mortality; therefore, targeted ultrasound screening for vasa previa is recommended, particularly in women with high-risk factors [10,12-14]. The UK National Screening Committee (UK NSC) advised that a national screening programme for vasa previa is not recommended because there is inadequate information on the number of babies affected by it in the UK [6,15]. RCOG also outlined that there is insufficient evidence to back up universal screening for vasa previa in routine foetal anomaly scans during mid-pregnancy in the general population [6]. Given type III vasa previa often occurs in the absence of recognised risk factors and is typically linked with a normal placenta and normal cord insertion, screening with transvaginal ultrasound and Doppler may be warranted in the general obstetric population during the first and second trimesters, with repeat assessment in high-risk women during the third trimester [4,12]. Therefore, it may be crucial to implement systematic screening using transvaginal ultrasound in pregnant women, especially in those who have a low-lying placenta or placenta structural abnormalities [4,12]. In addition, Ruiter et al. showed that the combination of colour Doppler and transvaginal ultrasound reported a high precision rate in detecting vasa previa prenatally, with a specificity of 99.0 to 99.8% and sensitivity of 100% [3]. Zhang et al. evaluated that the two-stage screening for vasa previa at 11 to 13 weeks and 20 to 22 weeks of gestation could potentially lower the stillbirth rate by approximately 10% [16]. While there is currently no universal screening programme for vasa previa, further research is required to investigate the role of screening for vasa previa in the general population and the cost-effectiveness of having screening for these patients because early detection is crucial, as it allows for elective caesarean section before rupture of membranes, thereby improving neonatal outcomes [6,14]. This patient was diagnosed at 23 weeks, and serial scans along with cervical length measurements were performed. Cervical length measurements using transvaginal ultrasound have been demonstrated to be inversely proportional to preterm rupture of membranes and preterm birth; hence, some studies have recommended that cervical length measurements may help predict the risk of foetal vessel rupture [17,18]. In the study by Zhang et al., 15 patients with vasa previa who underwent elective caesarean deliveries at 34 weeks exhibited cervical lengths above the fifth centile. In contrast, all five patients who required emergency caesarean section due to ruptured membranes or labour had cervical lengths below the fifth centile [16,18]. Oyelese et al. suggested measuring the cervical length in pregnant women with vasa previa every two to four weeks from the third trimester [18].

The RCOG guideline suggests considering prophylactic hospitalisation in women with vasa previa from 30 to 32 weeks of gestation, depending on individual risk factors [6]. In our case, the lady was hospitalised when she experienced PV bleeding at 24 weeks, followed by an elective admission around 33 weeks at her request. The decision for prophylactic hospitalisation should be individualised, considering a combination of factors including symptoms of contractions or bleeding, maternal and obstetric history, and logistical factors [2]. The American Journal of Obstetrics and Gynaecology (ACOG) recommends that further research is required on the role of hospitalisation amongst patients with vasa previa and the role of fetoscopic laser ablation as management for certain cases of vasa previa [14]. Furthermore, the RCOG guideline recommends elective caesarean section for asymptomatic women with vasa previa around 34-36 weeks [6]. This patient had a caesarean section performed at 35+5 weeks. This was decided based on the established diagnosis of vasa previa on serial scans and maternal anxiety, in the patient’s best interest. The patient was counselled antenatally regarding the diagnosis of vasa previa, including the risk of foetal hypoxia, exsanguination, preterm birth, and increased neonatal mortality if membranes rupture or labour occurs. The need for close monitoring, possible antenatal admission, and planned caesarean delivery was discussed. Norvilaitė K et al. reported a rare case of vasa previa at the Pregnancy Pathology Unit of Vilnius University Hospital Santariškių Clinics, where the patient had a Caesarean section at 33 weeks of pregnancy following hospitalisation with preterm rupture of membranes [19]. A female newborn was born weighing 2320 g; the APGAR score was 8 and 9 at 1 and 5 minutes, respectively [19]. The placenta was manually removed, revealing a membranous anchorage [19]. Early detection of vasa previa in this case enabled a multidisciplinary team involving the cardiologist, midwives, anaesthetist, and obstetrician to implement a safe care plan, culminating in a planned caesarean section at 35+5 weeks. This timing was chosen based on the confirmed diagnosis from serial scans and maternal anxiety, following the shared-decision process. This emphasises the importance of early detection of vasa previa to improve perinatal outcome, as well as considering different factors to formulate the management plan in the patient’s best interest. Overall, this case outlines early diagnosis and appropriate multidisciplinary management in a patient with vasa previa, which can pave the way for further research on screening, diagnosis, and management of vasa previa.

## Conclusions

Vasa previa is a rare but life-threatening condition that, if undiagnosed, can lead to heavy bleeding and foetal death. This case report highlights the importance of timely diagnosis and appropriate management in achieving favourable perinatal outcomes. 

While there is currently no universal screening programme for vasa previa, emerging evidence suggests that type III vasa previa may occur even in the presence of a normal placenta and cord insertion. Therefore, further research is needed into the implementation of systematic screening for vasa previa in the general pregnant population. Targeted screening for women with risk factors for vasa previa may offer a more cost-effective approach.

Outpatient or inpatient monitoring and the timing of delivery should be tailored according to patient wishes, gestation, ultrasound scan findings, and individual risk factors. Additionally, the integration of preterm clinics for dual screening of cervical shortening and vasa previa may enhance early detection rates. Overall, this case highlights the value of early diagnosis and comprehensive multidisciplinary management in improving perinatal outcomes.
